# Icariin Improves Stress Resistance and Extends Lifespan in *Caenorhabditis elegans* through *hsf-1* and *daf-2*-Driven Hormesis

**DOI:** 10.3390/ijms25010352

**Published:** 2023-12-26

**Authors:** Monika N. Todorova, Martina S. Savova, Liliya V. Mihaylova, Milen I. Georgiev

**Affiliations:** 1 Laboratory of Metabolomics, Institute of Microbiology, Bulgarian Academy of Sciences, 139 Ruski Blvd., 4000 Plovdiv, Bulgaria; mntodorova@yahoo.com (M.N.T.); m.sav@abv.bg (M.S.S.); liliya.vl.mihaylova@gmail.com (L.V.M.); 2 Department of Plant Cell Biotechnology, Center of Plant Systems Biology and Biotechnology, 4000 Plovdiv, Bulgaria

**Keywords:** longevity, aging, lifespan, healthspan, icariin, *Caenorhabditis elegans*

## Abstract

Aging presents an increasingly significant challenge globally, driven by the growing proportion of individuals aged 60 and older. Currently, there is substantial research interest in pro-longevity interventions that target pivotal signaling pathways, aiming not only to extend lifespan but also to enhance healthspan. One particularly promising approach involves inducing a hormetic response through the utilization of natural compounds defined as hormetins. Various studies have introduced the flavonoid icariin as beneficial for age-related diseases such as cardiovascular and neurodegenerative conditions. To validate its potential pro-longevity properties, we employed *Caenorhabditis elegans* as an experimental platform. The accumulated results suggest that icariin extends the lifespan of *C. elegans* through modulation of the DAF-2, corresponding to the insulin/IGF-1 signaling pathway in humans. Additionally, we identified increased resistance to heat and oxidative stress, modulation of lipid metabolism, improved late-life healthspan, and an extended lifespan upon icariin treatment. Consequently, a model mechanism of action was provided for icariin that involves the modulation of various players within the stress-response network. Collectively, the obtained data reveal that icariin is a potential hormetic agent with geroprotective properties that merits future developments.

## 1. Introduction

Aging is commonly referred to as a natural process accompanying the lifespan of a living being. However, throughout the evolution of aging and longevity research, along with the accumulated data over the years and the revolutionary scientific discoveries, the main concept of aging itself and its definition has changed [1,2,3]. Furthermore, the distinction between what constitutes aging and how we interpret it, either chronologically or biologically, has contributed to the expansion of this field of scientific research [1,4,5]. In theory, aging can be described as a gradual and cumulative loss of function in all organs and systems within the body machinery [6,7,8], commencing at the microenvironmental level, including phenomena like epigenetic changes, and ultimately culminating in the onset of age-related diseases. However, the question of whether we are all designed to age remains open. Longevity has been demonstrated as a possible avenue, as evidenced by centenarians who have proven that extended lifespan along with old-age healthspan is achievable [9].

Moreover, in an aging world where the proportion of people who reach beyond 60 years of age is constantly growing, improving healthspan has become a matter of great concern. In the past decade, dietary regimens such as fasting and caloric restriction, the use of pharmacological interventions such as senolytics therapies, and the use of natural small molecules like resveratrol, curcumin, and spermidine have been proven to increase lifespan and enhance the health status [2,3]. Furthermore, the unraveling of key molecular pathways implemented in longevity, such as the adenosine monophosphate protein kinase (AMPK), mammalian target of rapamycin (mTOR), insulin growth factor 1 (IGF-1), phosphoinositide 3 kinase (PI3K)/protein kinase B (AKT), and sirtuins (SIRTs) have directed the research endeavors to their pharmaceutical modulation [1,2,9].

Therefore, one potential approach towards age reversal is the pharmacological utilization of plant-derived natural compounds. Plants have been used for centuries not only as an existential source of nutrients but also for their medicinal properties, owing to the abundance of biologically active molecules they contain [9,10]. Hence, exploring the properties of secondary metabolites in the context of aging and age-related diseases is a promising avenue for longevity and healthspan research. Moreover, employing model organisms offers distinct advantages in studying the aging process and its potential reversibility. Such a model organism is *Caenorhabditis elegans* which allows valuable insights to be gained into the underlying mechanisms of aging and the testing of various interventions that could lead to enhanced healthspan and potentially longevity. Thus, *C. elegans* has been proven through years of research as an important platform for aging research due to its short lifespan and the availability of mutant strains with different phenotypes representing the comorbidities associated with aging. For instance, the long-lived strains that have mutations in evolutionarily conserved genes (such as *daf-2* that encodes insulin/IGF-1-like receptor, age-1 that corresponds to the PI3K, etc.) are associated with prolonged lifespan. The DAF-2-mediated longevity is strongly correlated to the activity of the DAF-16 transcription factor, which is the functional homolog of the Forkhead box O (FOXO) protein family [11,12,13].

Several studies have extensively explored the role of icariin as a naturally occurring flavonoid (most abundantly found in plants of the Epimedium genus) in inflammation processes, focusing on its ability to modulate inflammatory pathways and cytokine production [14]. Furthermore, icariin has been proven to be an atheroprotective and neuroprotective compound [15,16]. However, its potential involvement in aging and longevity remains relatively unexplored [17,18]. Some limited research suggests that icariin might play a role in regulating pro-longevity pathways [18,19,20]. Specifically, it has been proposed that icariin may impact components of these pathways, such as enhancing SIRT6 activity and influencing the nuclear factor erythroid 2-related factor 2 (NRF2) transcription factor [14,19,21]. Early studies investigating the bioactivity of icariin, particularly its derivative icariside II, have provided preliminary insights indicating that it may influence the DAF-2/insulin/IGF-1 signaling (IIS) pathway while also enhancing the healthspan in *C. elegans* [17]. Subsequently, a study in mice examined the lifespan-extending effect of icariin by maintaining the genome integrity [18]. Nevertheless, these findings are not sufficient to definitively elucidate the molecular network and mechanism of action behind icariin treatment.

To gain a more profound understanding of the intricate molecular framework, we employed the *daf-2* mutant strain, which features an inhibited IIS pathway resulting in a doubled lifespan, allowing for a comparison with the wild-type *C. elegans*. In addition, our investigation assessed whether icariin could influence the worms’ responsiveness to stressors, covering two distinct time points in their life cycle that represent different stages of aging. Our research utilized a multifaceted methodology to investigate the impact of the flavonoid icariin on healthspan and lifespan in *C. elegans*. We conducted a gene expression analysis of pro-longevity pathways that are evolutionarily conserved between worms and mammals, with the aim of gaining comprehensive insights into their participation in the mechanism of action of icariin.

## 2. Results

### 2.1. Icariin Prolongs the Lifespan and Improves the Fitness of Wild-Type C. elegans in Comparison to daf-2 Loss-of-Function Mutant

**Figure 1:**
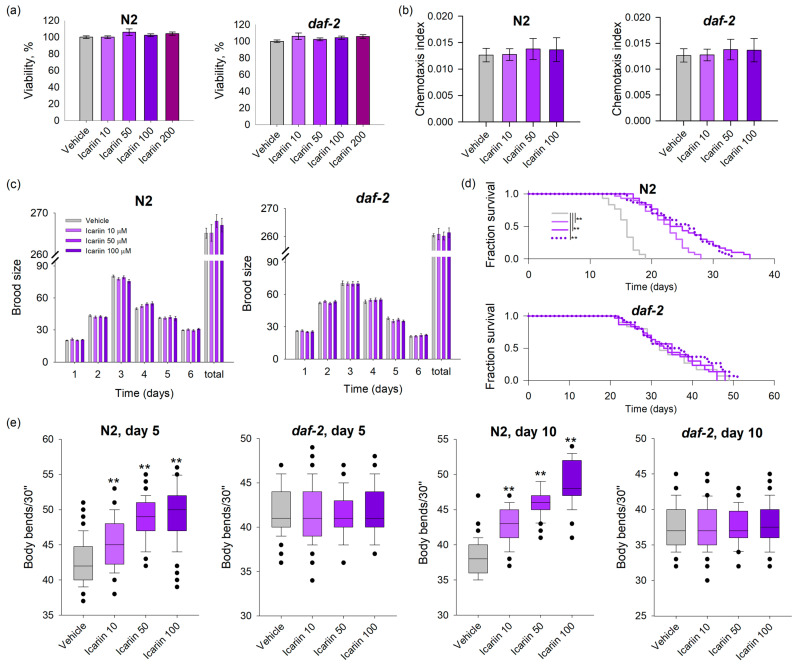
Icariin treatment extends the lifespan and enhances the mobility of wild-type *C. elegans* in contrast to *daf-2* mutant strain. (**a**) Viability test upon icariin treatment (10–200 μM) in N2 and *daf-2 C. elegans*. The statistical significance of the survival curves was assessed using the Log-rank test. (**b**) Chemotaxis assay of icariin-treated *C. elegans* (n = 300–600) from both strains used. (**c**) Daily and total brood size of icariin-treated N2 and *daf-2* nematodes (n = 15). (**d**) The comparison of the lifespan between icariin-treated worms and the vehicle group was performed using a Kaplan–Meier survival curve represented as a fraction survival (n = 90). (**e**) Numbers of body bends in 30 s on both day 5 and day 10 in both strains used (n = 45). Data for (**b**–**e**) are presented as mean ± SEM, ** *p* < 0.01 compared to the vehicle group by one-way analysis of variance (ANOVA).

To assess the safety of icariin on nematodes, a viability assay was conducted [22]. During the larval stages (L1-L4), nematodes were subjected to a 48 h incubation period with icariin at concentrations ranging from 10 to 200 μM (Figure 1a). Following this exposure, viability was scored, revealing that the selected doses were non-toxic for both wild-type and mutant strains. Based on this preliminary outcome, concentrations of 10, 50, and 100 μM were selected for subsequent assays.

To evaluate the impact of icariin on the worms’ physiology, phenotypic assays were performed to observe any potential changes or alterations. The chemotaxis behavior provides insights into the neurosensory system and serves as a physiological indicator of the worms’ feeding preferences and the avoidance of toxic chemicals [23]. Chemosensation in *C. elegans* is associated with the neuroendocrine network and its participants, such as DAF-7, which corresponds to the mammalian transforming growth factor beta (TGF-β), which is known for its role in dauer larva formation [24]. To determine the effect of icariin on the chemosensory neural network of the nematodes, we conducted a chemotaxis assay. The results showed that N2 and *daf-2* mutant nematodes neither exhibited a higher attraction to the studied concentrations of icariin nor did they avoid it, compared to the control group (Figure 1b). Interpreting these results as a physiological response, the nematodes’ neutral reaction to icariin during the one-hour analysis period may suggest that the substance does not affect the neuroendocrine network, at least not within the specified time frame, and confirms its safety.

The reproductive cycle of an organism is often considered a representation of its healthspan, and *C. elegans* does not deviate from this pattern [25]. External factors and the supplementation of compounds, whether plant-derived or synthetic, can influence the organism’s physiological processes and may impact important functions such as reproduction. Furthermore, reproductive fitness decline is a common trait of aging in *C. elegans* [26]. Therefore, the daily and total brood size of icariin-treated worms was evaluated and compared to the vehicle group. Our results show that the nematodes’ fertility remained unaffected by the treatment (Figure 1c). Along with a lack of effect on reproductive fitness, no disturbances or abnormalities were observed during the nematodes’ reproductive cycle under the applied icariin concentrations. Hence, it is reasonable to conclude that icariin has no adverse effects on the reproductive capacity of *C. elegans* wild-type and *daf-2* loss-of-function mutants (Figure 1c).

In regard to the icariin effect on *C. elegans* lifespan, all three concentrations employed induced a significant lifespan extension in N2 wild-type worms. In contrast, treatment with icariin in the long-lived mutant strain *daf-2* did not result in lifespan improvement (Figure 1d). Hence, the observed ability of icariin to enhance the survival of N2 worms offers evidence supporting the involvement of DAF-2/insulin/IGF receptor signaling in icariin-mediated longevity.

Senescence-related changes have implications for the motility of worms [12]. Studies indicate that aging in worms is linked to progressive deterioration and functional decline in muscle mass, along with neuropathological outcomes. These events are comparable to human late-lifespan diseases, such as Alzheimer’s and Parkinson’s disease, that are associated with uncoordinated or reduced movements [12,27]. Assessing the rate of bending throughout a worm’s lifespan serves as one of the reliable indicators of its healthspan. An increased lifespan, as seen in the long-lived mutant strain such as *daf-2(e1370)*, does not always correlate with an improved healthspan. These worms exhibit a frailty period and a decline in trashing rate similar to that observed in wild-type worms, as demonstrated by Bansal et al. [27]. To examine the effects of icariin on the locomotion of *C. elegans*, we selected two timepoints in the worms’ lifespan—day 5 and day 10. Measurements of bending movements at both timepoints revealed that icariin significantly improved locomotion in the N2 strain in a dose-dependent manner compared to the vehicle (Figure 1e). Conversely, icariin treatment had no impact on the motility of *daf-2* worms. This contrast suggests that the mechanism through which icariin influences worm motility during aging correlates to the lifespan extension data and is dependent on the *daf-2* signaling pathway.

Obtained data suggest that improved mobility and increased lifespan upon icariin treatment are attributed to modulation in *daf-2* signaling.

### 2.2. Icariin Ameliorates Healthspan in C. elegans via Enhanced Response to Stressors

Increased longevity in *C. elegans* is often associated with enhanced resistance to various stressors [28,29,30]. Therefore, based on our results showing lifespan improvement due to icariin treatment, we conducted additional stress assays to explore the compound’s potential to improve worm heat and oxidative stress resistance.

**Figure 2:**
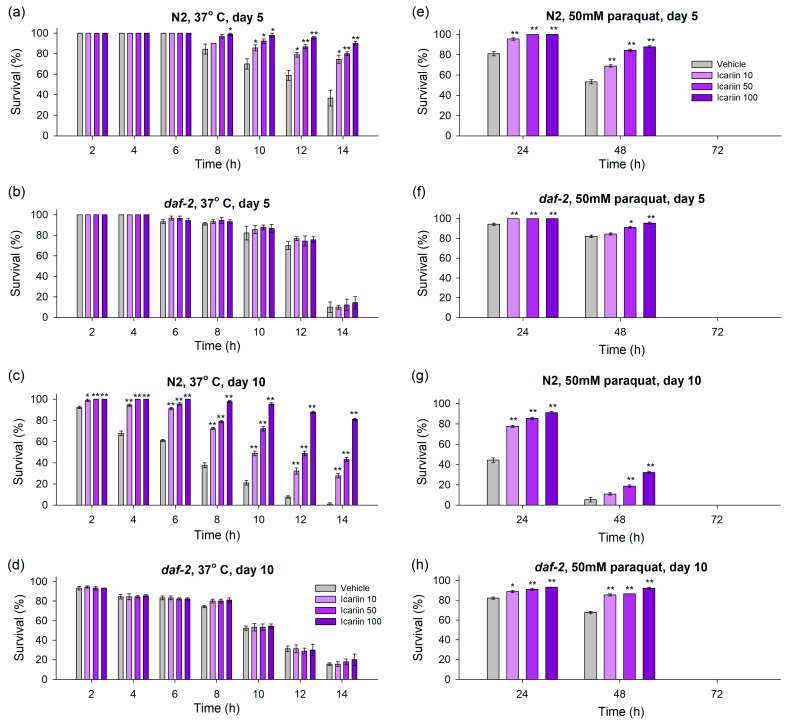
Icariin treatment enhances heat and oxidative stress resistance in *C. elegans*. Wild-type N2 (**a**,**c**,**e**,**g**) and *daf-2* mutant (**b**,**d**,**f**,**h**) strains were subjected to treatment with icariin at concentrations of 10, 50, and 100 μM. (**a**–**d**) For heat-stress induction, on both the 5th and 10th days of their lifespan, they were incubated at 37 °C for 14 h. The viability was monitored at two-hour intervals until the death of the last worm. (**e**–**h**) As an oxidative stressor, on day 5 and day 10 of their lifespan, the worms were exposed to a paraquat concentration of 50 mM. The resistance to oxidative stress was assessed at 24 h intervals until the death of the last worm. The results were represented as percentage survival as mean ± SEM, n = 90, * *p* < 0.05, ** *p* < 0.01 (one-way ANOVA).

To assess thermotolerance, we exposed *C. elegans* pre-treated with icariin to a temperature of 37 °C and monitored their survival rate every two hours during a fourteen-hour time window (Figure 2a–d).

Furthermore, given that organisms often experience a decline in their ability to mitigate stressors with age, we conducted the assay on day 5 of the worms’ lifespan, when initial aging signs appear, and on day 10, when aging processes become more pronounced. Regarding the wild-type, icariin, applied in all three concentrations, has strengthened the thermotolerance on both day 5 and day 10 of the worms’ lifespan (Figure 2a,c). Previous studies have assessed that *daf-2* mutants display increased resistance to stressors [29]. However, the sensitivity to thermal stimuli of these mutants is still a controversial subject [27]. The pre-treatment of the *daf-2* mutants with icariin did not result in an improvement in heat resistance in selected timepoints (Figure 2b,d). This effect could also be attributed to the involvement of the IIS pathway, as evidenced by the fact that the heat resistance of *daf-2* mutants was not improved by icariin.

Many pro-longevity interventions, such as natural compound supplementation or dietary regimens, increase the resilience to stressors, including oxidative stress burden. This effect is underpinned by the activation of stress defense systems and their key players, including SIRT1 and SIRT6 (SIR-2.1 and SIR-2.4 in *C. elegans*, respectively), the master regulator of oxidative stress response NRF2 (SKN-1 in nematodes) and the heat shock proteins (HSF-1 in *C. elegans*) [9,31]. Exposure to high levels of toxic agents, such as paraquat or juglone, has been proven to drastically reduce worm lifespan and lead to lethality [30,32]. Therefore, to assess whether icariin could enhance *C. elegans*’ resilience to oxidative stress, we treated the nematodes with a high dose of paraquat (50 mM) and monitored their viability every 24 h till the dead of the last worm (Figure 2e–h).

Our results provide compelling evidence of a significant enhancement in stress resistance through icariin supplementation. On the 5th day of their lifespan, wild-type worms exhibited a clear dose-dependent increase in oxidative resistance compared to the control group, as indicated by monitoring at both 24 and 48 h. On the 10th day, the benefits of icariin remained evident among worms tested 24 h after stress exposure. However, by the 48 h mark, significant endurance to paraquat was observed only at concentrations of 50 and 100 μM (Figure 2g).

An intriguing observation arose in the *daf-2* mutant groups, recognized for their increased survival to mild stress. Analyzing the temporal aspect (days 5 and 10), all three concentrations of icariin substantially improved stress resistance 24 h after acute paraquat stress induction (Figure 2f,h). An exception was observed at the 48th hour on day 5 when only concentrations of 50 and 100 μM demonstrated the capacity to enhance stress resistance in the *daf-2* mutant worms compared to the control group (Figure 2f,h). It is important to note, however, that icariin did not confer protection against the accumulated toxicity over time and eventual lethality. All worms exposed to paraquat did not survive beyond the 72 h mark of monitoring (Figure 2e–h).

Collectively, our data suggest that icariin’s health-enhancing and stress-endurance effect may arise from a mechanism operating in parallel to the *daf-2*-dependent IIS pathway. This mechanism seems to be specifically activated in response to oxidative stress, as opposed to heat stress, given that our results showed improved thermotolerance only in wild-type worms.

### 2.3. Icariin Decreases the Lipid Droplet Accumulation

**Figure 3:**
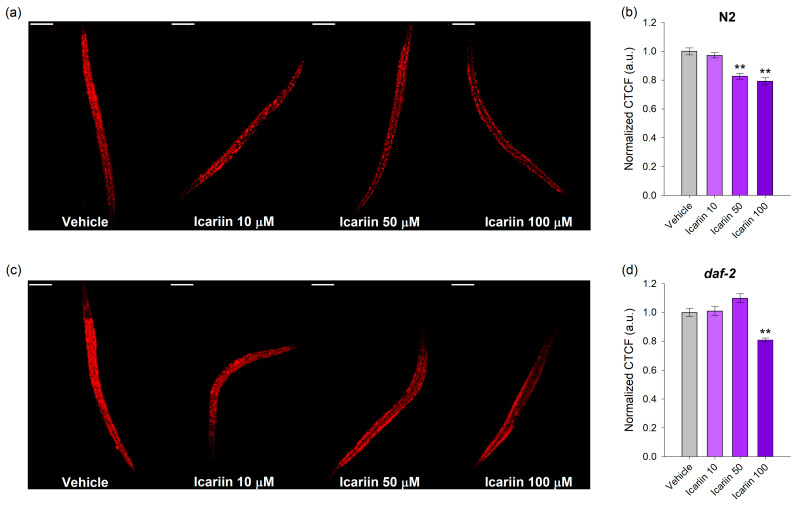
Icariin supplementation modulates fat metabolism in *C. elegans*. Representative confocal photographs at 20× magnification (scale bar of 50 μm) of Nile red lipid staining of wild type (**a**) and *daf-2* mutant strain (**c**) nematodes treated for 24 h with 10, 50, and 100 μM icariin or vehicle. Quantification of lipid accumulation as normalized correlated total cell fluorescence (n = 90), corrected total cell fluorescence (CTCF) expressed as arbitrary units (a.u.) as follows for wild-type (**b**) and *daf-2* (**d**) nematodes. Values are shown as mean ± SEM, ** *p* < 0.01 compared to the vehicle group (one-way ANOVA).

The obtained data from the phenotypic and stress assays served as the foundation for our investigation into the potential impact of icariin on nematode lipid accumulation. Locomotor activity not only serves as an indicator of the absence of degeneration in the nervous and muscular systems but also signifies heightened energy expenditure [31,32,33]. Additionally, the combination of increased resistance to oxidative and thermal stress displayed by the worms, coupled with enhanced body bending, is frequently associated with the concept of hormesis [34,35]. This phenomenon forms the basis of various pro-longevity interventions, encompassing fasting, dietary restriction, and controlled exposure to stressors [34,35,36,37]. Furthermore, studies conducted on model organisms report the existence of plant-derived molecules that mimic the effects of mild stressors, such as dietary restriction, resulting in reduced lipid accumulation concurrently with improved stress resistance and subsequent extension of lifespan [10]. Hence, our objective was to investigate whether the administration of icariin to nematodes within a 48 h timeframe could influence their lipid metabolism. Icariin supplementation in wild-type *C. elegans* in 50 and 100 μM concentrations significantly decreased the total lipid content (Figure 3a,b). In general, the *daf-2* mutants are characterized by elevated lipid accumulation due to their restricted IIS signaling [29]. Correspondingly, in the *daf-2* mutants, a higher concentration of icariin (100 μM) is required for lipid reduction (Figure 3c,d).

Our results provide insights into the *daf-2*-dependent lipid-reducing effect of icariin, particularly evident at the higher concentrations. Moreover, icariin may exert broader physiological effects, potentially modulating various molecular pathways beyond the scope of *daf-2*. These findings expand our understanding of icariin’s impact on lipid metabolism in nematodes and underscore the complexity of its potential mechanisms of action.

### 2.4. Icariin Alters the Transcriptional Regulation of Evolutionarily Conserved Genes Involved in Stress Resistance and Lifespan Regulation

To further explore the molecular network associated with the beneficial effects of icariin on lifespan, we conducted a comprehensive investigation of the daf-2/IIS pathway, along with an in-depth examination of essential components within the molecular network associated with longevity, stress resistance, and nutrient sensing, including *skn-1*, *sir-2.1*, *sir-2.4*, and *hsf-1* [38].

**Figure 4:**
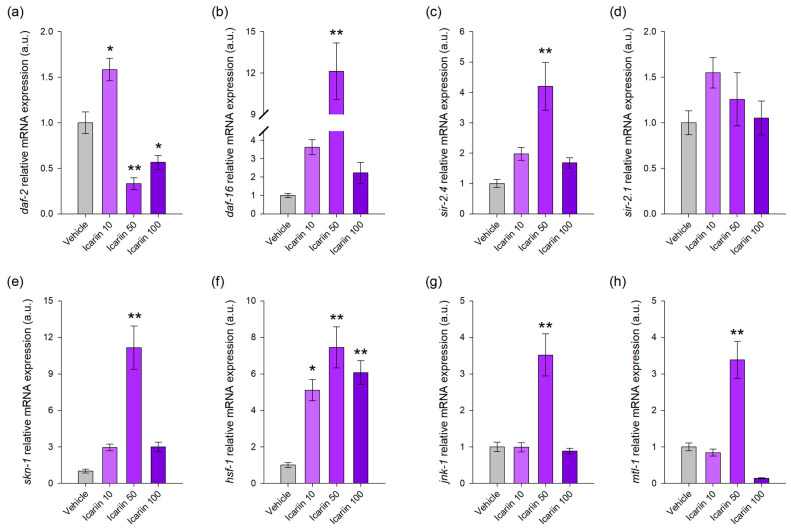
Icariin treatment modulates gene expression of evolutionarily conserved pro-longevity pathways in wild-type *C. elegans*. Normalized relative mRNA expression in arbitrary units (a.u.) of *daf-2* (**a**), *daf-16* (**b**), *sir-2.4* (**c**), *sir-2.1* (**d**), *skn-1* (**e**), *hsf-1* (**f**), *jnk-1* (**g**), and *mtl-1* (**h**) upon treatment with 10, 50, and 100 μM icariin. Data shown are mean ± SEM, n = 9. * *p* < 0.05, ** *p* < 0.01 compared to the vehicle group (one-way ANOVA).

The transcriptional analysis results provided further mechanistic information for our hypothesis, demonstrating that icariin mediates *daf-2* inhibition and regulates lifespan through the IIS pathway. Specifically, we observed different responses induced by the three experimental concentrations. Icariin, when applied at concentrations of 50 μM and 100 μM, induced the repression of *daf-2* mRNA levels (Figure 4a), resulting in the activation of distinct mechanisms for each concentration. At a concentration of 50 μM, *daf-2* inhibition correlated with increased expression of *daf-16* (Figure 4b) and its target gene metallothionein (*mtl-1*; Figure 4h), along with activation of Jun N-terminal kinase (*jnk-1*; Figure 4g), a known regulator of *daf-16* in response to stressors [38]. Additionally, upon 50 μM icariin treatment, *skn-1* (Figure 4e) and *hsf-1* (Figure 4f) exhibited higher transcriptional activity, and are regulators acting in parallel with daf-16 to induce longevity [39]. Interestingly, *sir-2.4* (Figure 4c) but not *sir-2.1* (Figure 4d) was detected with an upregulated expression pattern in the result of icariin treatment. At a concentration of 100 μM, icariin induced significant upregulation only in *hsf-1* (Figure 4e), with no substantial alterations detected in the expression of the other studied genes, which is the gene’s distinct response to icariin at this concentration.

Taken together, the gene expression analysis defined *hsf-1* as a major player in the icariin effect, acting in parallel with *daf-2* and *daf-16* in the concentration of 50 μM and solely in the concentration of 100 μM applied.

### 2.5. Icariin Regulates DAF-16 Subcellular Localization

Building upon the gene expression results and the critical role of DAF-16 in regulating aging processes and longevity, our focus was to determine whether icariin treatment could impact its nuclear translocation in the *daf-16(ot971[daf-16::GFP])* mutant strain.

**Figure 5:**
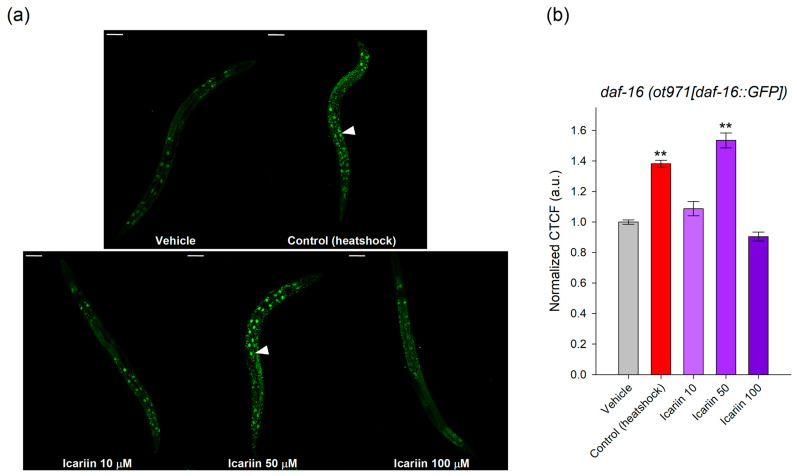
Nuclear translocation of DAF-16 was observed upon icariin treatment. Evaluation of nuclear translocation of *daf-16(ot971[daf-16::GFP])* mutant strain. (**a**) Representative fluorescent confocal microphotographs at 20× magnification (scale bar of 50 μm) of vehicle, heat-shocked control (37 °C for 5 min) or icariin-treated *C. elegans daf-16(ot971[daf-16::GFP])*. Arrowheads indicate increased nuclear localization. (**b**) Using ImageJ version 1.53t, the fluorescent signal was processed to cell total correlates fluorescence (CTCF), normalized to the vehicle group, and represented in arbitrary units (a.u.). The data are shown as means ± SEM, n = 60, ** *p* < 0.01, compared to the vehicle (one-way ANOVA).

Intriguingly, our findings revealed that icariin at a concentration of 50 μM induced translocation to the nucleus of the *daf-16* tagged allele, compared to the vehicle (Figure 5b), as evidenced by the confocal photographs (Figure 5a). These findings correspond to the gene expression results (Figure 4b), further supporting our suggestion that the mechanism of action of icariin at a concentration of 50 μM is dependent on DAF-16 activity.

### 2.6. Model Mechanism Representing the Hormetic Effect of Icariin in C. elegans

**Figure 6:**
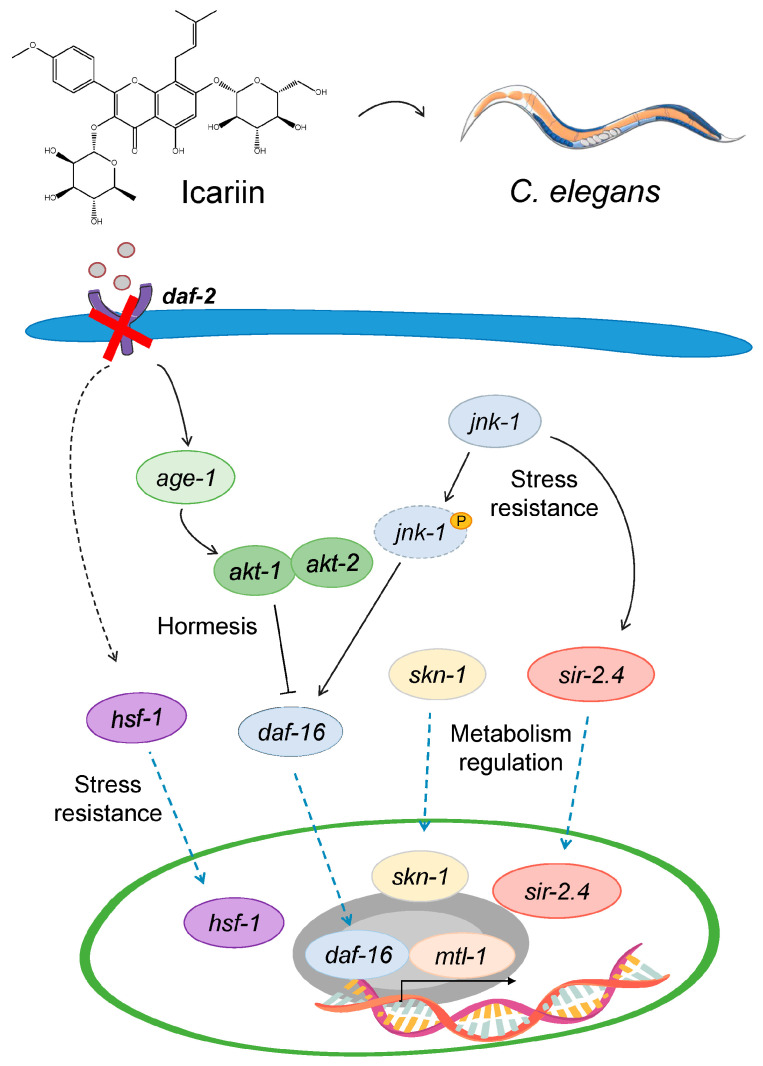
Mechanism-based model representing the hormetic response of icariin to extend lifespan in *C. elegans*. At a concentration of 50 μM, icariin inhibits the *daf-2* gene that triggers a cascade of events within the IIS pathway. Specifically, DAF-16 translocates to the nucleus, where it activates the expression of *mtl-1*, a gene responsible for encoding metallothionein, which is a critical player in the organism’s response to stressors. Under stressful conditions, our observations suggest a potential involvement of *jnk-1* in facilitating the nuclear translocation of DAF-16, along with possible concurrent activation of *sir-2.4*. Furthermore, upregulation of *skn-1* may potentially contribute to improvements in overall fitness and healthspan. Notably, the *hsf-1* is overexpressed by icariin at all treatment concentrations that expose it as a main contributor to the observed lifespan-extending activity.

The collected results provide evidence that conserved longevity and stress–response associated molecular mechanisms are involved in the biological activity of icariin in *C. elegans*. A schematic representation of the proposed mechanism-based model is provided in Figure 6.

## 3. Discussion

Longevity regulation involves evolutionarily conserved genes and pathways related to nutrient and energy-sensing molecular networks. Key components include the mTOR, which influences cellular growth and autophagy in response to amino acids and carbohydrates availability, and the AMPK, which responds to increased AMP and ADP levels, triggering autophagy and an oxidative stress response. Sirtuins (e.g., SIRT1-7) sense NAD+ levels and interact with IIS, AMPK, FOXO, and mTOR signaling to extend lifespan [2,9]. These mechanisms are evolutionarily conserved across species, emphasizing the genetic control over aging. However, they can be modulated by interventions such as lifestyle modifications, dietary changes, or pharmacological approaches like targeted drug therapy [9]. Studies also indicate that mild exposure to stress conditions, such as brief cold-temperature exposure or moderate oxygen deprivation, could be beneficial for organisms—a phenomenon termed hormesis. This activation of stress–response mechanisms, on the other hand, increases healthspan [40,41]. The first signaling pathway associated with longevity and healthspan, uncovering the involvement of the deep molecular network behind aging, was the insulin/IGF-1/FOXO pathway [42]. As an ortholog of the human insulin/IGF-1 receptor, DAF-2 in *C. elegans* also plays a role in aging and longevity [43,44,45]. Functional DAF-2 triggers a series of downstream reactions, including the phosphorylation-driven activation of AKT-1/2. Consequently, AKT inhibits DAF-16, retaining it within the cytoplasm. Diminishing IIS activity in worms, exemplified by inhibition of DAF-2 pathway through geroprotectors or pro-longevity interventions, leads to the translocation of DAF-16 into the nucleus, ultimately governing the transcription of its target genes and promoting healthspan [43,44,45]. However, other pivotal transcription factors participate in DAF-2-mediated longevity, including SKN-1 and HSF-1 [43]. Targeting the IGF receptor in mice, even later in life, has been demonstrated to yield beneficial effects on health and its improvement while also increasing the potential for extended longevity [46]. In contrast, in the context of humans, the landscape is more intricate, with a mix of conflicting findings regarding the insulin and IGF-1 pathways [47]. Defects in insulin receptor signaling lead to insulin resistance and diabetes, while certain genetic variations that decrease signaling in these pathways are linked to enhanced longevity, particularly in centenarian populations [48,49,50]. Interestingly, deficiencies in IGF-1 or growth hormone are associated with an increased risk of cardiovascular disease, yet centenarians tend to exhibit improved insulin sensitivity, low IGF-1 levels, and specific mutations in the insulin receptor that are associated with extreme longevity [51,52]. In contrast to the IIS pathway, the FOXO family of transcription factors is well known for serving as a central hub in enhancing cellular resistance to a wide range of stressors, including oxidative, metabolic, and replicative stress responses [38]. In centenarians, FOXO3 and its genetic variations have been specifically linked to longevity [48,52,53]. This underscores the critical role of FOXO3 in translating signals from the IIS pathway into alterations in the gene expression profiles of its targeted genes. Furthermore, FOXO3 plays a crucial role in regulating nutrient sensing, autophagic processes, and preserving DNA integrity when faced with diverse stressors [38]. In summary, FOXO3 emerges as an evolutionarily conserved regulatory circuit with a substantial impact on lifespan [38]. In *C. elegans,* DAF-16 plays a supportive role in longevity as a downstream target of DAF-2 [39]. Moreover, DAF-16 can integrate signals from other molecular axes that run parallel to the IIS pathway.

Our results indicate the involvement of both *daf-2* and DAF-16 in icariin-mediated longevity, along with other players of the conserved pro-longevity networks such as *skn-1*, *hsf-1*, and *sir-2.4*. The lifespan of wild-type worms was significantly extended through icariin treatment, but the compound did not have the same effect on the *daf-2* loss-of-function mutant. This finding was the first indication in our study of the involvement of the IIS pathway in the mechanism of action of icariin. Multiple indicators, including improved heat-stress tolerance and oxidative stress resistance in the wild-type strain, as well as enhanced locomotion in response to icariin treatment at both timepoints during the worm’s lifespan, suggest improved healthspan and a delay in the age-related degradation of the neuronal circuitry and muscles. Furthermore, the increased motility could be associated with an elevated energy expenditure [33,54], a characteristic often associated with pro-longevity interventions such as caloric restriction and fasting. In worms, food deprivation for starvation-induced longevity is also accompanied by improved locomotion, enhanced lipid metabolism, and reduced IIS activity. Additionally, many studies suggest that progressive lipid droplet accumulation during aging leads to lipotoxicity and disrupted lipid homeostasis [55]. Examination of fat depositions through Nile red staining and confocal imaging after icariin treatment further illustrated that icariin exhibits a *daf-2*-dependent lipid-reducing effect. Interestingly, icariin also mediated lipid droplet depletion in the *daf-2* loss-of-function strain, but this effect was observed only at a concentration of 100 µM. These results indicate that icariin may affect parallel pathways together with the DAF-2 to modulate lipid homeostasis, which could also involve processes like autophagosome-lysosome activity [56].

The beneficial effects of pro-longevity interventions that target lipid metabolism, such as dietary regimens and exercise, have been associated with the concept of hormesis [40,41]. Hormesis in aging suggests that moderate stress can confer physiological benefits by activating protective mechanisms within cells and organisms. This response to stressors, whether physical, physiological, biological, or nutritional, triggers a cascade of processes that influence adaptation and longevity, collectively referred to as the stress response [40,41,57]. In *C. elegans*, various types of stressors induce hormesis, interacting with the metabolome, transcriptome, and proteome, respectively. Brief exposure to heat shock during early adulthood extends the lifespan of worms and enhances resistance to high levels of stressors [57]. Hypoxic stress in worms is another example of hormesis, particularly induced in mild doses and associated with the gustatory sensory system [58]. On the other hand, dietary phytochemicals such as resveratrol, curcumin, and quercetin, along with certain vitamins and minerals, also have the ability to induce mild stress responses in model organisms [35,59]. These compounds hold promise as potential anti-aging agents, partly attributed to their capacity to activate the nutrient-sensing network through a hormetic-like effect [10].

To verify our hypothesis that icariin may exhibit hormetic activity, we examined the worms’ response to two types of stressors: heat stress and oxidative stress. Our results showed that icariin enhances resilience to both stressors in wild-type worms during both early and late-life adulthood. The absence of survival in both N2 and daf-2 worms after 72 h of paraquat treatment could likely be attributed to the use of a high dose of paraquat (50 mM). The administration of such a concentrated dose induces acute stress and leads to lethality after approximately 24 h [60,61]. Surprisingly, the long-lived *daf-2* strain also exhibited icariin-mediated improvements in resistance to acute paraquat treatment, especially at the 50 and 100 µM treatments. This strain is known to have enhanced resistance to mild oxidative stress due to its loss-of-function *daf-2* mutation [29]. However, our results suggest that another stress-induced pathway could be activated, recruiting the organism’s survival mechanisms. This initiation of responses could be attributed to the heat shock response, unfolded protein response, and DNA damage response [59], further indicating that icariin may act as a hormetic agent. A substantial body of evidence has highlighted that hormetic-like phytochemicals activate more than one key pathway, including nuclear factor-erythroid 2, sirtuins, nuclear factor-kappa B, and the heat shock response [35,43,59,62].

Natural compounds have demonstrated the ability to activate the molecular network associated with the phenomenon of hormesis, thereby modulating HSF-1 and the molecular network regulated by this transcription factor [40,41]. Heat shock factor 1 is evolutionarily conserved across species and serves as the master regulator behind the induction of hormesis. Its modulation is associated with certain late-life diseases linked to aging, such as metabolic disturbances and obesity. The HSF-1 is an evolutionarily conserved protein linked to the response to different stressors [57,63]. Furthermore, HSF-1 is associated with the maintenance of proteostasis integrity and the regulation of mitochondrial function during the aging process [64]. These findings hint at a promising role for *hsf-1* in the modulation of the hormetic-like response, contributing to the enhancement of healthspan and longevity that merits further validation.

Biologically active molecules often manifest biphasic dose–response patterns, whereby low and high doses elicit distinct signaling pathways [62]. This phenomenon is observed not only in well-established hormetic compounds such as quercetin, sulforaphane, capsaicin, epigallocatechin gallate, and resveratrol [35,43,59,62] but also in the case of icariin as our results suggest. The hormetic response induced by such natural compounds is often associated with an optimal dose range, where the natural compound continues to exert beneficial effects, reaching the peak stimulation of certain molecular pathways [35,43,59]. Our findings indicate that icariin adheres to this hormetic dose–response pattern, revealing discernible signaling pathways engaged depending on the concentration applied. Furthermore, *skn-1* is a pivotal transcription factor associated with the hormetic effect, prominently implicated in the response mediated by icariin at a concentration of 50 μM. The activation of SKN-1 has been linked not only to the mTOR signaling pathway, responding to amino acid and carbohydrate scarcity but also to the downregulation of DAF-2 and the activation of DAF-16. Research indicates a collaborative role for DAF-16 and SKN-1 as transcriptional regulators that enhance lysosomal activity and integrity throughout the aging process, crucial for sustaining healthspan and extending lifespan [56]. Both *skn-1* and *daf-16* are upregulated in response to icariin treatment at a 50 μM concentration, and only this concentration induces DAF-16 nuclear translocation. Additionally, the activation of *sir-2.4* by icariin, along with the activation of its mediator, *jnk-1*, aligns with previous studies suggesting their involvement in response to stress conditions, particularly oxidative stress [46,65]. In contrast, when the dose is increased to 100 μM, only *hsf-1* is transcriptionally active. This corresponds to a surpassed optimal range of the treatment, which exerts an inhibitory effect on some of the molecular pathways involved in the effects of the lower dose used [35,41,59]. Thus, we could hypothesize that icariin has a hormetic-like nature and exerts its effects through a wide array of molecular targets, dependent on the applied concentration. In summary, our research has illuminated the potential of icariin as a promising hormetic agent capable of mimicking mild stress conditions to enhance healthspan. Our results have unveiled its capacity to enhance genome and proteome integrity throughout the aging process, thereby improving stress resistance and overall fitness. Moreover, the intricate molecular network involved in its mechanism of action positions icariin as a powerful modulator of multiple age-related pathologies. By regulating various aging mechanisms and improving the healthspan, icariin emerges as a compelling candidate for promoting longevity and overall well-being. The challenge ahead lies in precisely defining the doses associated with its pro-longevity effects and delving even deeper into the elucidation of the modulated mechanisms. In conclusion, our study has proposed a mechanism-based model of the lifespan-extending effect of icariin through *hsf-1* and *daf-2*-driven hormesis that ratifies its further examinations as a potential geroprotector.

## 4. Materials and Methods

### 4.1. Materials

Icariin (molecular weight 676.66 g/M; purity ≥ 99.06%; Cat. № HY-N0014) is supplied from MedChemExpress (Sollentuna, Sweden). Nematode growth medium (NGM; Cat. № MBS652667) was purchased from MyBiosource Inc. (San Diego, CA, USA). The LB broth Lennox (Cat. № L3022), agar powder (Cat. № 05039), M9 minimal salts (Cat. № M6030), 3-(4,5-dimethylthiazol-2-yl)-2,5-diphenyl tetrazolium bromide (MTT; Cat. № M2128), fluoroshield histology mounting medium (Cat. № F6182), Nile red (NR, Cat. № 72485), sodium hydroxide, and paraquat (purity ≥ 98%) were purchased from Sigma-Aldrich (St. Louis, MO, USA). Reagents and consumables for RNA isolation, gel electrophoresis, and quantitative real-time polymerase chain reaction (RT-qPCR) analyses were supplied by Bio-Rad Laboratories Inc. (Hercules, CA, USA).

### 4.2. Caenorhabditis Elegans Maintenance and Treatment

Strains used in this study were obtained by the Caenorhabditis Genetic Centre (CGC, University of Minnesota, MN, USA, which is funded by NIH Office of Research Infrastructure Programs (P40 OD010440). The nematodes were grown according to standard procedures on NGM plates seeded with *Escherichia coli* OP50 as a food source. The *C. elegans* strains used were as follows: wild-type Bristol strain N2, *CB1370 daf-2(e1370)* III, OH16024 *daf-16(ot971[daf-16::GFP])* I. For the following experiments, a standard hypochlorite bleaching method was used to obtain an age-synchronized worm population. The experimental treatment was added to heat-inactivated and concentrated 10-fold *E. coli* OP50 at final concentrations of 10, 50, and 100 μM. The experimental concentrations were selected following evaluation of the viability of the N2 and *daf-2* nematodes for 48 h by MMT-based assay [22].

### 4.3. Fecundity Assay

For estimation of the impact of icariin on worm reproductive capacity, a fecundity assay was performed. At least five nematodes from each experimental group were randomly selected and individually transferred to a plate NGM seeded with inactivated *E. coli* OP50, supplemented with icariin (10, 50, and 100 μM). After reaching the fertility stage, the number of laid eggs from every individual was counted daily until the end of the reproductive period. The experiments were carried out in triplicates.

### 4.4. Locomotion Assay

Worms were transferred to the NGM plates and incubated with icariin at concentrations 10, 50, and 100 μM for 5 and 10 days. Evaluation of the bending movements of the worms was performed as previously described [28,64]. The locomotion was measured on day 5 and day 10 of the worm’s lifespan. Nematodes from all experimental groups at both timepoints were randomly picked and placed in a drop of M9 buffer on a clear NGM plate, allowing the worms to adapt for 30 sec. Therefore, the number of body bends in 30 s was observed under a stereomicroscope from KERN & SOHN GmbH (Balingen, Germany). The assay was performed in triplicates, and at least 15 worms were used as representative fragments for each group.

### 4.5. Lifespan Measurement

Lifespan measurements were performed following previously established protocols [66,67]. Around 30 synchronized late L4 larvae were randomly selected and transferred to NGM plates seeded with inactivated *E. coli* OP50 containing icariin. This day was defined as day 0 of the lifespan. The worms were monitored daily for survival or compromised health and were subsequently transferred daily until the end of the reproduction cycle. After the end of the reproduction cycle, they were moved to fresh NGM plates containing the tested compound and OP50 every other day, respectively. Nematodes that did not respond to external mechanical stimuli, such as a platinum touch, were considered dead. Additionally, individuals that crawled out of the plate were censored and excluded from further analyses. Details about mean and maximum lifespan are provided in Supplementary Table S1.

### 4.6. Chemotaxis

The chemotaxis assay was performed as previously described with a slight modification [68]. The petri dish was divided into four quadrants, which were designated as the test (“T”) or control (“C”) zone. A volume of 2 μL of the control and the test sample were placed in the corresponding quadrant. A circle with 1 cm diameter was drawn in the center of the petri dish, in which about 100–150 L4 nematodes (in a drop of 2 μL of M9 buffer) were placed (Supplementary Figure S1). After an incubation time of 1 h at 20 °C, the petri dish was placed at 4–6 °C for 30 min to immobilize the nematodes, facilitating their counting. Nematodes in each quadrant of the petri were counted, and the chemotaxis index (CI) was calculated based on the formula: CI = (Quadrant test area 1 + Quadrant test area 2) − (Quadrant control area 1 + Quadrant control area 2)/Total number of nematodes.

### 4.7. Nile Red Triglyceride Staining

The staining of fat depositions in *C. elegans* was evaluated using the Nile red assay. The staining procedure was carried out according to the previously described method [54,69]. Briefly, after 24 h of treatment with icariin at different concentrations, approximately 1000–1500 L4 larvae were collected and washed three times with M9 buffer. After each wash, the larvae were centrifuged, and the supernatant was removed. The worm pellet was then fixed with 40% isopropanol for 3 min at room temperature. After removing the fixative, the staining solution was applied, and the worms were incubated for two hours. The imaging of the lipid depositions was performed using the confocal system Stellaris 5 with an inverted microscope DMi8 from Leica (Wetzlar, Germany). The quantification of fluorescence intensity was performed using ImageJ software version 1.53t normalized to the vehicle group. The results were presented as normalized CTCF in arbitrary units (a.u.).

### 4.8. Heat Stress

The heat stress resistance analysis involved a minimum of 30 animals in each group, and the experiments were independently repeated at least three times. Synchronized populations of N2 and daf-2 late L4 larvae or young adults (n ≥ 100) were maintained in standard conditions on NGM plates, supplemented with icariin (10, 50, and 100 μM) until the nematodes reached day 5 and day 10 of adulthood. At both selected timepoints, the temperature was upshifted to 37 °C for 14 h [70]. The dead individuals were identified by gently touching them with a platinum wire pick and were scored every 2 h to monitor the survival rate.

### 4.9. Oxidative Stress

For the oxidative stress resistance assay, at least 30 nematodes pre-treated with icariin were transferred to fresh NGM plates containing 50 mM paraquat [71] on both the 5th and 10th days of their lifespan. The worms were subjected to daily monitoring, and the deceased ones were recorded at 24 h intervals until the death of the last one. The experiment was conducted in triplicate, with each experimental group consisting of over 30 nematodes. The results were presented as percentage survival for each timepoint of measurement.

### 4.10. Gene Expression Analysis through RT-qPCR

Total RNA was extracted with PureZol (Bio-Rad) from around 3000–4000 L4 wild-type nematodes and treated for 24 h with icariin or vehicle. Agarose gel electrophoresis and UV spectroscopy were used to determine the integrity and quantity of the extracted RNA. Reverse transcription for mRNAs was performed using the First strand cDNA synthesis kit (Canvax, Cordoba, Spain). The expression of mRNAs was quantified by ΔΔCT method on the CFX Maestro software version 4.1.2433.1219 Bio-Rad Laboratories Inc. (Hercules, CA, USA). The *iscu-1* and *mdh-1* were used for endogenous control for mRNAs, and the results were normalized to the vehicle group [28]. The results were presented as normalized relative mRNA expression in arbitrary units. Nucleotide sequences of the primers, used for analysis of relative mRNA expression, are provided in Table 1.

### 4.11. Detection of DAF-16 Nuclear Translocation

To further investigate the involvement of DAF-16 transcriptional activity, we examined whether icariin treatment induces cytoplasmic-to-nuclear translocation. For this purpose, we utilized the mutant strain OH16024 *daf-16(ot971[daf-16::GFP])*, which features fluorophore-tagged *daf-16*. Synchronized L3 larvae were exposed to icariin treatment for 24 h. Subsequently, the nematodes were transferred to 2% agarose pads, and levamisole solution was administered to induce anesthesia. To serve as a positive control for nuclear localization, a brief heat stress was applied to the group of worms at 37 °C for 5 min. The experiment was conducted in triplicate, with each experimental group consisting of over 20 nematodes. The expression and cellular localization of the GFP-tagged allele were examined through fluorescence microscopy [72,73]. Mean fluorescence intensities were quantified using ImageJ as CTCF and normalized to the vehicle group. The results were presented as normalized CTCF in arbitrary units.

### 4.12. Statistical Analysis

Statistical analyses were performed in SigmaPlot version 11.0 from Systat software GmbH (Erkrath, Germany), and the data were represented as mean ± SEM. Variations between the experimental groups were calculated by one-way ANOVA, followed by Tukey’s post hoc test. The level of statistical significance was set at * *p* < 0.05 and ** *p* < 0.01. The Kaplan–Meier survival curves of the different groups were compared using the log-rank test to assess the statistical significance between them. The experimental data presented are representative of at least three independent biological experiments.

## Figures and Tables

**Table 1:** Primer sequences used in the RT-qPCR of mRNAs.

Gene	Forward	Reverse
*daf-2*	GAGACACGATGCGAGTGAGACG	GGATCAGCGGCTTCTTTCCACC
*daf-16*	TCCGTCCCCGAACTCAATCGAACC	TGCTGGAACCGATTCGCCAACC
*sir-2.4*	TTATCGGAGCCGGTGTGAGCAC	GGGACGAGCAACTTGAAAGTCCAC
*sir-2.1*	TGTGTTTGTTTCGGGTGCATCGG	AGAAGTTGCGGTCACACACGGG
*skn-1*	TTCCGCGTCGACGAATCTTGCG	AGCTTCCAGTGTCGGCGTTCCA
*hsf-1*	TCATCGTGGGATTTCTGCGCTG	CCATTCTTGCTCCAGCTCCAGC
*jnk-1*	TCCTGTGCACATGGAGGAACGA	AGGAGGATGCGAGCAGAGTGTT
*mtl-1*	TGCAGTGGAGACAAGTGTTGTG	GCTCTGCACAATGACAGTTTGC
*mdh-1*	GGACCATTCATCGCCACTGTCC	TGTGATCACAAGCGGCCTTAGC
*iscu-1*	CTCCTGCACAAGTTTGCGTTGC	CCGACGCTTGGATCGTTCTTGT

## Data Availability

All relevant data are within the manuscript. The data set generated and analyzed during the current study is also available from the corresponding author upon request.

## Supplementary Materials

The supporting information can be downloaded at: https://www.mdpi.com/article/10.3390/ijms25010352/s1.
