# Supplementary material for: Icariin Improves Stress Resistance and Extends Lifespan in Caenorhabditis elegans through hsf-1 and daf-2-Driven Hormesis
Source: Int J Mol Sci. 2023 Dec 26;25(1):352. doi: 10.3390/ijms25010352 (PMC10778813; doi:10.3390/ijms25010352)
Supplement: Supplementary file 1 [file ijms-25-00352-s001.zip › ijms-2768431-supplementary.pdf]

Supplementary information

Icariin Improves Stress Resistance and Extends Lifespan in *Caenorhabditis elegans* Through *hsf-1* and *daf-2*-driven Hormesis

Monika N. Todorova[1], Martina S. Savova[1,2], Liliya V. Mihaylova[1,2] and Milen I. Georgiev[1,2,*]

1 Laboratory of Metabolomics, Institute of Microbiology, Bulgarian Academy of Sciences, 139 Ruski Blvd., 4000 Plovdiv, Bulgaria; mntodorova@yahoo.com; m.sav@abv.bg; liliya.vl.mihaylova@gmail.com; milengeorgiev@gbg.bg

2 Department of Plant Cell Biotechnology, Center of Plant Systems Biology and Biotechnology, 4000 Plovdiv, Bulgaria; m.sav@abv.bg; liliya.vl.mihaylova@gmail.com; milengeorgiev@gbg.bg

* Correspondence: milengeorgiev@gbg.bg; Tel: 00359 32 64 24 30

Supplementary Figures

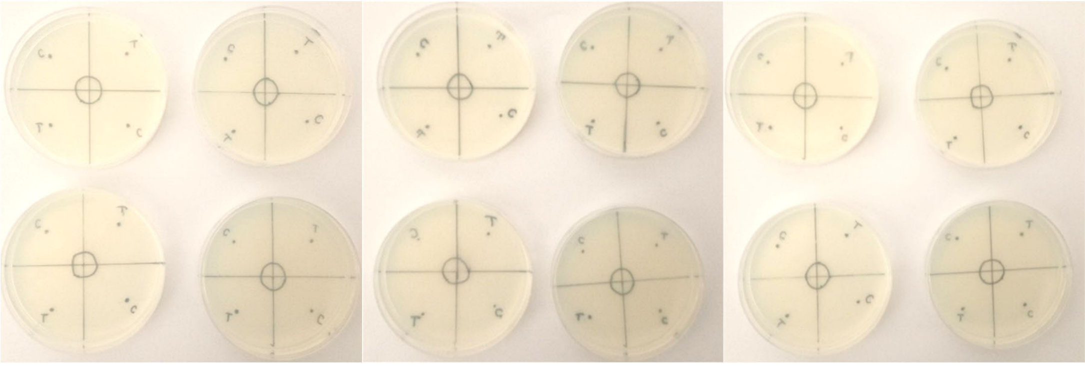

Supplementary Figure S1. Images of assaying plates used for chemotaxis evaluation.

Supplementary Tables

Supplementary Table S1. Effect of icariin treatment (10, 50 and 100 μM) on mean and maximum lifespan in days of wild-type and *daf-2* mutant *C. elegans*.

Strain	Treatment	Mean lifespan (SEM)	Maximum lifespan (SEM)
N2 (wild type)	Vehicle	15.76 (0.23)	19.78 (0.28)
	Icariin 10	22.17 (0.39)	27.78 (0.27)
	Icariin 50	25.2 (0.61)	35.67 (0.33)
	Icariin 100	24.83 (0.57)	32.75 (0.26)
daf-2 (e1370)	Vehicle	34 (0.86)	48.33 (0.41)
	Icariin 10	35.1 (0.89)	48.44 (0.24)
	Icariin 50	34.29 (0.84)	46.56 (0.24)
	Icariin 100	36.33 (1)	50.56 (0.34)
